# Analysis of the genetic diversity of the nematode parasite *Baylisascaris schroederi* from wild giant pandas in different mountain ranges in China

**DOI:** 10.1186/1756-3305-6-233

**Published:** 2013-08-08

**Authors:** Xuan Zhou, Yue Xie, Zhi-he Zhang, Cheng-dong Wang, Yun Sun, Xiao-bin Gu, Shu-xian Wang, Xue-rong Peng, Guang-you Yang

**Affiliations:** 1 Department of Parasitology, College of Veterinary Medicine, Sichuan Agricultural University, Ya’an 625014, China; 2 Chengdu Research Base of Giant Panda Breeding, Chengdu 610081, China; 3 Department of Chemistry, College of Life and Basic Science, Sichuan Agricultural University, Ya’an 625014, China

**Keywords:** Giant panda, *Baylisascaris schroederi*, Mountain ranges, Genetic diversity, Genetic structure, Phylogeography

## Abstract

**Background:**

*Baylisascaris schroederi* is one of the most common nematodes of the giant panda, and can cause severe baylisascarosis in both wild and captive giant pandas. Previous studies of the giant pandas indicated that this population is genetically distinct, implying the presence of a new subspecies. Based on the co-evolution between the parasite and the host, the aim of this study was to investigate the genetic differentiation in the *B*. *schroederi* population collected from giant pandas inhabiting different mountain ranges, and further to identify whether the evolution of this parasite correlates with the evolution of giant pandas.

**Methods:**

In this study, 48 *B*. *schroederi* were collected from 28 wild giant pandas inhabiting the Qinling, Minshan and Qionglai mountain ranges in China. The complete sequence of the mitochondrial cytochrome b (mt*Cytb*) gene was amplified by PCR, and the corresponding population genetic diversity of the three mountain populations was determined. In addition, we discussed the evolutionary relationship between *B*. *schroederi* and its host giant panda.

**Results:**

For the DNA dataset, insignificant Fst values and a significant, high level of gene flow were detected among the three mountain populations of *B*. *schroederi*, and high genetic variation within populations and a low genetic distance were observed. Both phylogenetic analyses and network mapping of the 16 haplotypes revealed a dispersed pattern and an absence of branches strictly corresponding to the three mountain range sampling sites. Neutrality tests and mismatch analysis indicated that *B*. *schroederi* experienced a population expansion in the past.

**Conclusions:**

Taken together, the dispersed haplotype map, extremely high gene flow among the three populations of *B*. *schroederi*, low genetic structure and rapid evolutionary rate suggest that the *B*. *schroederi* populations did not follow a pattern of isolation by distance, indicating the existence of physical connections before these populations became geographically separated.

## Background

The giant panda, one of the world’s most iconic and threatened species, is considered a precious natural resource [1-3]. Giant pandas are currently restricted to the Qinling, Minshan, Qionglai, Daxiangling, Xiaoxiangling and Liangshan Mountains on the Eastern edge of the Tibetan plateau, and number about 1,600 individuals, of which 17.23% are in Qinling, 44.36% in Minshan and 27.38% in Qionglai [4,5]. *Baylisascaris schroederi* is one of the most common nematodes of giant panda, and can cause severe baylisascarosis in both wild and captive giant pandas [1,2,6]. The parasite mainly lives in the small intestine of the giant panda. However, the migrating *B*. *schroederi* larvae are sometimes also recovered post-mortem from the liver, lungs, heart or brain, which is associated with an increased clinical severity and pathologic manifestations, making *B*. *schroederi* one of the most serious parasites of giant panda [2].

Understanding organismal biodiversity based on the analysis of genetic variation has important implications for studying the evolutionary history and genetic structure of populations, and may therefore provide basic data for disease control. In general, genetic diversity, population hierarchical structure, population mutation rate, rate of gene flow and selective neutrality are the quantifiable components of genetic structure [7]. Parasitic nematodes are unexceptional to this universal biological rule [7]. During recent decades, a significant amount of genetic data has been generated on nematode parasite populations, in an attempt to explain micro-evolutionary processes [8]. However, unlike most free-living organisms, not only could the parasites own reproductive and transmission patterns but also their host genetics and behavior (e.g. migration) influence the genetic variations in parasites. Thus, genetic markers can be used to understand and depict the population genetic structure and diversity of a number of animal nematodes [8].

Previous studies of the giant pandas in the Qinling Mountain range indicated that this population is distinct from other wild giant panda populations, implying the presence of a new subspecies [9]. However, recent investigations based on whole genome data analysis have indicated that the Minshan and Qionglai-Daxiangling-Xiaoxiangling-Liangshan population were also genetically distinct [10]. In general, the genetic correlations between a population of hosts and their parasites are usually consistent, due to their similar demographic history and local adaptations. In this study, we investigated whether there is geographical genetic differentiation within these populations, and further identified whether the evolution of this parasite correlates with the evolution of the giant panda. The complete mt*Cytb* gene (1107 bp) as a genetic marker in 48 isolates of *B*. *schroederi* parasitizing 28 pandas inhabiting three different mountain ranges (Qinling, Minshan and Qionglai) was sequenced. Based on these samples, the genetic diversity and geographical relationships of the *B*. *shroederi* populations were examined in the three relatively isolated mountain ranges, which the giant panda mainly inhabits.

## Methods

### Ethics statement

The present study was performed in strict accordance with the Guidelines and Recommendations for the Care and Use of Laboratory Animals of the Ministry of Health of the People’s Republic of China, and all protocols were reviewed and approved by the Research Ethics Committee of Sichuan Agricultural University (Ya’an, China).

### Sample collection, identification and DNA extraction

**Figure 1:**
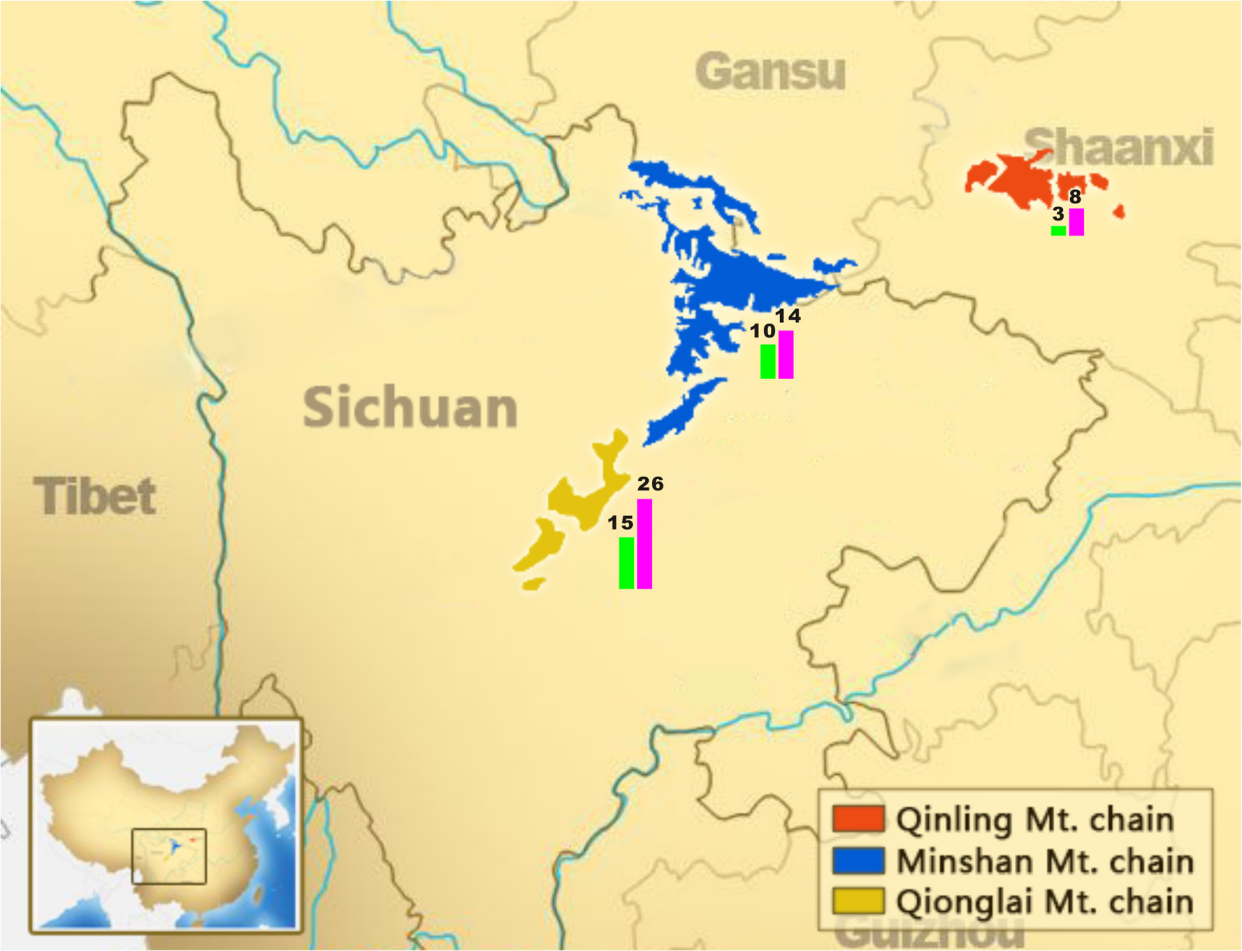
**Map of the sampling sites.** The geographical location of *B*. *schroederi* isolates collected from three different mountain ranges in China. The numbers of *B*. *schroederi* isolates (purple bars) and sampled hosts (green bars) from each mountain are shown respectively.

Adult *B*. *schroederi* (n = 48) were collected from 28 dead, injured or rescued giant pandas from three mountain ranges (Qinling, Minshan and Qionglai) during the period between May 2000 and May 2012 (details in Additional file 1; Figure 1). Individual worms were washed in physiological saline, identified morphologically as *B*. *schroederi*[6], and stored at −20°C before DNA extraction. Total nematode genomic DNA was extracted from each specimen by standard proteinase K treatment and phenol/chloroform extraction [11], eluted into 30 μL TE buffer (pH 8.0) and stored at −20°C until analysis.

### Polymerase chain reaction (PCR) amplification, cloning and DNA sequencing

Using the published mtDNA sequence of *B*. *schroederi* in GenBank (HQ671081) [1], the primers Cytb-1 (GGTGCTATGCTCGGTTACG) and Cytb-2 (CCACTAAGACCCTCCATT) were designed to amplify the whole mt*Cytb* gene (~1500 bp) from the 48 nematode specimens. The optimal cycling conditions for specific and efficient amplification were obtained by testing varying annealing temperatures. PCR reactions (25 μL) containing 1 μL of template genomic DNA, 1 μL of each primer (10 pmol each), 12.5 μL of 2× Taq MasterMix (Beijing ComWin Biotech, Beijing, China), 9.5 μL of ddH2O were performed in a S1000 Thermal Cycler (Bio-Rad, USA) using the following conditions: initial denaturation at 94°C for 5 min; 35 cycles of 94°C for 1 min (denaturation), 50°C for 1 min (annealing), 72°C for 1 min (extension); followed by a final extension at 72°C for 10 min. The amplifed PCR products were visualized on ethidium bromide-stained 1.0% agarose-TAE gels under UV light, excised and purified using spin columns (Wizard PCR Prep, Promega, USA). The purified products were cloned into the vector pMD19-T (TakaRa, Dalian, China) using standard molecular procedures. Each clone was sequenced three times on an automatic DNA sequencer (ABI Applied Biosystems Model 3730) by Invitrogen (Shanghai, China).

### Sequence alignment and phylogenetic analyses

All sequences were initially aligned using ClustalX 1.81 [12] with the following parameters: gap opening penalty = 10.0, gap extension penalty = 5.0, and then the alignments were modified by eye. A consensus phylogeny of the mtDNA haplotypes were estimated by two methods: maximum-parsimony (MP) tree using MEGA 5.10 [13] with the significance of each node estimated using 10000 bootstrap replicates of the data set, and the Bayesian inference (BI) using MrBayes 3.1 [14]. Four independent Markov chains were simultaneously run for the 1,630,000 replicates by sampling one tree per 1000 replicates with the Bayesian procedure. The first 250 trees were discarded as part of a burn-in procedure, and the remaining samples were used to generate a 50% majority rule consensus tree. *Baylisascaris transfuga* (accession number: NC_015924.1 for the mt*Cytb* gene), a closely related species [1], was used as the outgroup in these phylogenies.

### Population differentiation

The program Dna SP 5.0 [15] was used to calculate the nucleotide diversity within populations, number of informative sites, neutrality test (Fu’s *Fs* and Tajima’s D value), average number of nucleotide differences (K), nucleotide divergence (Dxy) and the net genetic distance (Da) between populations; the Fst statistic and value were computed from the haplotype frequencies using the program Arlequin Version 3.5 [16]. To evaluate whether genetic differentiation between populations was associated with geographical isolation of the mountains, analysis of molecular variance (AMOVA) [16] was used to examine genetic structuring among the sequences of the populations from the three mountain ranges: (i) Qinling Mountain range; (ii) Minshan Mountain range; (iii) Qionglai Mountain range. Gene flow was also estimated using Dna SP 5.0 [14].

The pairwise genetic distances between individual haplotypes were calculated following the Kimura 2-parameter model, using MEGA 5.0 [17]; phylogenetic relationships were estimated using Arlequin Version 3.5 [16] and then implemented in Network 4.6 [18] to illustrate the genetic relationships among the Cytb gene haplotypes in *B*. *schroederi* parasitizing giant pandas in three different mountain ranges.

### Population expansions

For population expansion analysis, the Arlequin Version 3.5 [16] coupled with 1000 simulations was used to obtain the distribution of Fu’s Fs value of neutrality and *P* value for each population, to assess whether population expansion occurred in *B*. *schroederi*. To calculate the distribution of the number of pairwise differences between the 48 sample sequences, a mismatch analysis was conducted using Dna SP 5.0 [15] over 1000 simulations.

### Estimation of time differentiation

Due to the lack of fossil records for *B*. *schroederi*, calibration of the absolute rate of evolution of these parasites is generally problematic. Therefore, the evolutionary rate of the mt*Cytb* gene from the parasite nematode *Heligmosomoides polygyrus*, (which belongs to the Nematoda, the same as *B*. *schroederi* (3.5-3.7% K_2_P distance per million years) [19] in this study), was used to estimate the mean Kimura 2-parameter (K_2_P) distance between the 48 parasite mt*Cytb* sequences in order to date the isolation time of *B*. *schroederi*.

## Results

### Haplotype and haplotype distribution

The complete sequence (1107 bp) of the mt*Cytb* in 48 *B*. *schroederi* isolates was determined and deposited in GenBank under the accession numbers KC796955-KC797002. The sequence analysis showed 31 variable sites on the sequences (2.80%), including 24 singleton variable sites, defining 16 haplotypes (Additional file 2). Nucleotide diversity in the 48 samples was 0.201%, and nucleotide diversity among the populations isolated in the different mountain ranges ranged from 0.1489% (Minshan) to 0.222% (Qionglai). The Minshan population had the lowest diversity (0.1489 ± 0.1046%). The number of haplotypes, haplotype diversity and nucleotide diversity of each population are presented in Table 1.

**Table 1:** **Summary of the genetic diversity of the three populations of*B***. ***schroederi*collected from giant pandas inhabiting different mountain ranges**

**Populations**	**No. individual**	**No. of haplotypes**	**No. of variable sites**	**Haplotype diversity (±SD)**	**Nucleotide diversity (±SD)**	**Tajima’s D**	**Fu’s Fs**	**Average no. of nucleotide differences (k)**
Minshan	14	7	10	0.7582 ± 0.1158	0.001489 ± 0.001046	−1.85262***	−2.69374*	1.648
Qinling	8	3	8	0.6786 ± 0.1220	0.002065 ± 0.001438	−1.25337	1.87372	2.286
Qionglai	26	12	21	0.8985 ± 0.0327	0.002221 ± 0.001382	−1.99313***	−4.91076***	2.458
Total	48	16	31	0.8440 ± 0.0378	0.002009 ± 0.001252	−1.69971*	−7.72261**	2.224

*P < 0.05; **P < 0.01; ***P < 0.001.

*SD* standard deviation.

### Phylogenetic relationships

**Figure 2:**
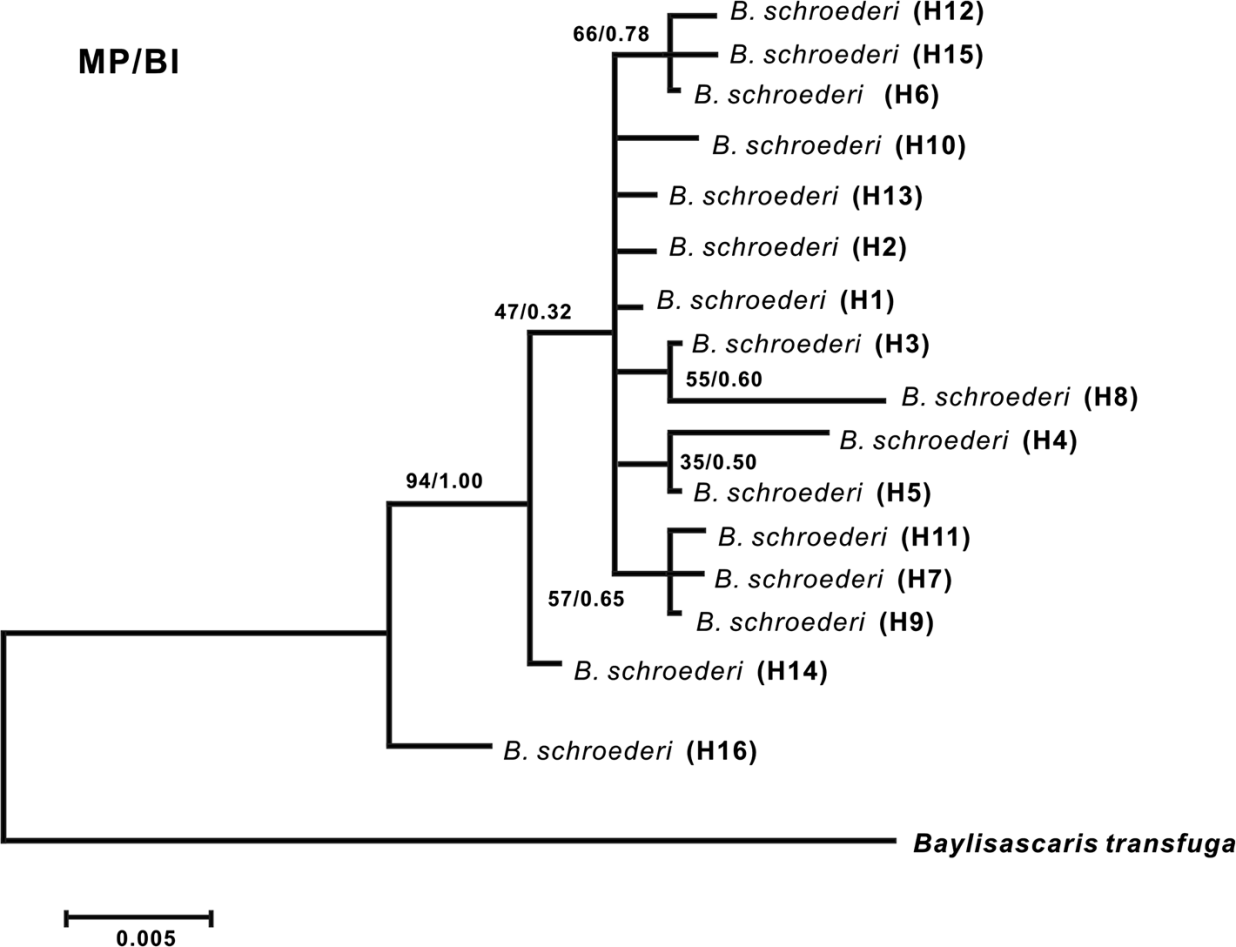
**MP and BI trees for the 16 mt*Cytb *haplotypes.** Maximum-parsimony, MP; Bayesian inference, BI. The numbers along branches indicate bootstrap values from different analyses in the order: MP/BI. *B*. *transfuga* as the outgroup.

**Figure 3:**
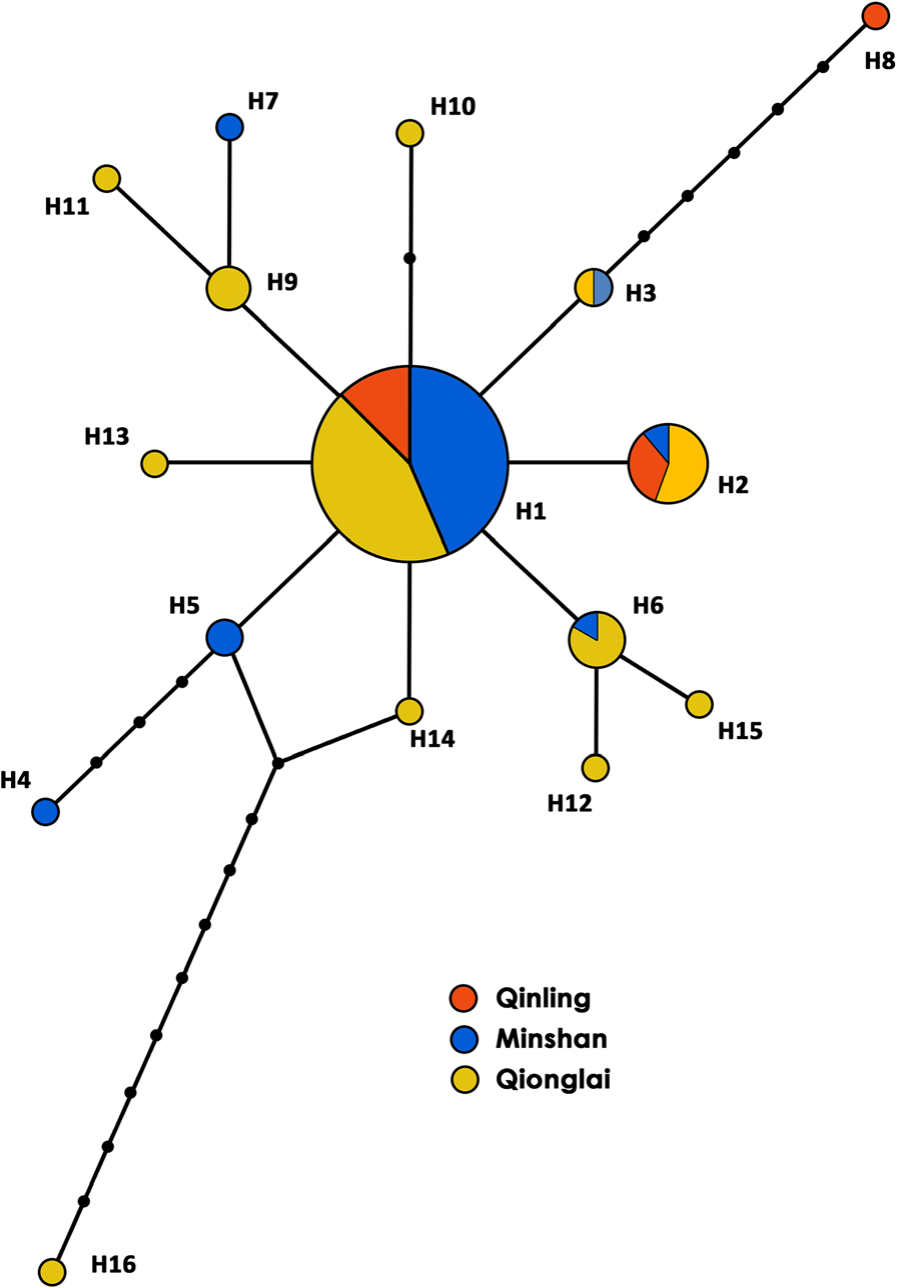
**Network map of the 16 mt*Cytb *haplotypes in *B.schroederi. *** The area of each circle is proportional to the haplotype frequency.

To clarify the phylogenetic relationship among the haplotypes of the three populations of *B*. *schroederi*, the sequence divergence values to construct hypothetical phylogenetic trees were calculated by the MP method and BI procedure. The two methods of phylogenetic analysis using the mt*Cytb* marker led to very similar trees with shallow branches (Figure 2). The topology of the resultant trees was supported by the bootstrap values. Interestingly, as shown in Figure 2, haplotype H7, which was specific to the Minshan population, closely clustered with haplotypes H9 and H11 in the Qionglai population; while the Minshan population-specific haplotype H3 was close to the Qinling population-specific haplotype H8; three haplotypes in the Qionglai population (H6, H12 and H15) clustered together; the other haplotypes formed isolated clusters. Several relationships among these haplotypes were also confirmed using the Network method (Figure 3). On the basis of the 16 haplotypes detected for the mt*Cytb* gene, the network map revealed a star-like pattern around haplotype H1 (16 individuals, 33.3% of total haplotypes). Seven of the haplotypes were found in the Minshan population (H1-H7), three in the Qinling population (H1, H2, H8) and twelve in the Qionglai population (H1-H3, H6, H9-H16). While H1 and H2 were present in the populations from all three mountain ranges, haplotype H1, which had a cumulative frequency of 33.33%, was more frequent than H2 (18.8%). The Qionglai and Minshan populations shared haplotypes H3 and H6 (Figure 3). Furthermore, the haplotypes present in each mountain population were highly dispersed, and no obvious correlation of sampled clusters was detected.

### Population genetic structure

To analyze the population genetic structure, several AMOVA analyses at different hierarchical levels were performed. Based on these AMOVA analyses, 98.03% of the variation occurred within the individual populations, and only 1.97% occurred across the three populations. As shown in Table 2, the highest *Fst* value was recorded for the Qinling and Qionglai populations (*Fst* = 0.02875, *P* > 0.05), while the lowest value was recorded for the Minshan and Qionglai populations (*Fst* = 0.01911, *P* > 0.05). The pairwise genetic distances between haplotypes are presented in Table 3; the pairwise genetic distances between haplotypes were generally low, except for H16 compared with H8 (1.7%), with a mean overall haplotype distance of 0.4% K_2_P. Nucleotide divergence (Dxy) among the three populations was low, ranging from 0.182% to 0.221%. Taken together, the three mountain range populations were not significantly differentiated.

**Table 2:** **Pairwise comparisons based on parameters in the three*B***. ***schroederi*populations**

**Population 1**	**Population 2**	**Nm**	**Fst**	**Dxy**	**Da**
Minshan	Qinling	9.68 ^a^	0.02519 ^a^	0.00182	0.00005
Minshan	Qionglai	12.83 ^a^	0.01911 ^a^	0.00189	0.00004
Qionglai	Qinling	8.45 ^a^	0.02875 ^a^	0.00221	0.00006

Nm, pairwise comparisons based on gene flow; Fst, genetic variance contained in a subpopulation relative to the total genetic variance; Dxy, nucleotide divergence; Da, net genetic distance for the three populations of *B*. *schroederi* collected from giant pandas inhabiting different mountain ranges; a, no significance.

**Table 3:** **Pairwise comparison of genetic distance and the percentage of haplotypes for the*B***. ***schroederi*population**

	**H1**	**H2**	**H3**	**H4**	**H5**	**H6**	**H7**	**H8**	**H9**	**H10**	**H11**	**H12**	**H13**	**H14**	**H15**	**H16**
	33.33%(n=16)	18.75% (n=9)	4.17% (n=2)	2.08% (n=1)	4.17% (n=2)	12.50% (n=6)	2.08% (n=1)	2.08% (n=1)	6.25% (n=3)	2.08% (n=1)	2.08% (n=1)	2.08% (n=1)	2.08% (n=1)	2.08% (n=1)	2.08% (n=1)	2.08% (n=1)
H1	-															
H2	0.001															
H3	0.001	0.002														
H4	0.005	0.005	0.005													
H5	0.001	0.002	0.002	0.004												
H6	0.001	0.002	0.002	0.005	0.002											
H7	0.002	0.003	0.003	0.006	0.003	0.003										
H8	0.006	0.007	0.005	0.009	0.007	0.007	0.008									
H9	0.001	0.002	0.002	0.005	0.002	0.002	0.001	0.007								
H10	0.002	0.003	0.003	0.006	0.003	0.003	0.004	0.008	0.003							
H11	0.002	0.003	0.003	0.006	0.003	0.003	0.002	0.008	0.001	0.004						
H12	0.002	0.003	0.003	0.006	0.003	0.001	0.004	0.008	0.003	0.004	0.004					
H13	0.002	0.002	0.002	0.005	0.003	0.002	0.003	0.007	0.002	0.003	0.003	0.004				
H14	0.001	0.002	0.002	0.005	0.003	0.002	0.003	0.007	0.002	0.003	0.003	0.003	0.002			
H15	0.002	0.003	0.003	0.006	0.003	0.001	0.004	0.008	0.003	0.004	0.004	0.002	0.003	0.003		
H16	0.01	0.011	0.011	0.013	0.003	0.011	0.012	0.017	0.011	0.012	0.012	0.012	0.011	0.009	0.012	-

Note: pairwise comparison of genetic distance is below diagonal and the percentage of haplotypes is above the diagonal.

### Population expansion

**Figure 4:**
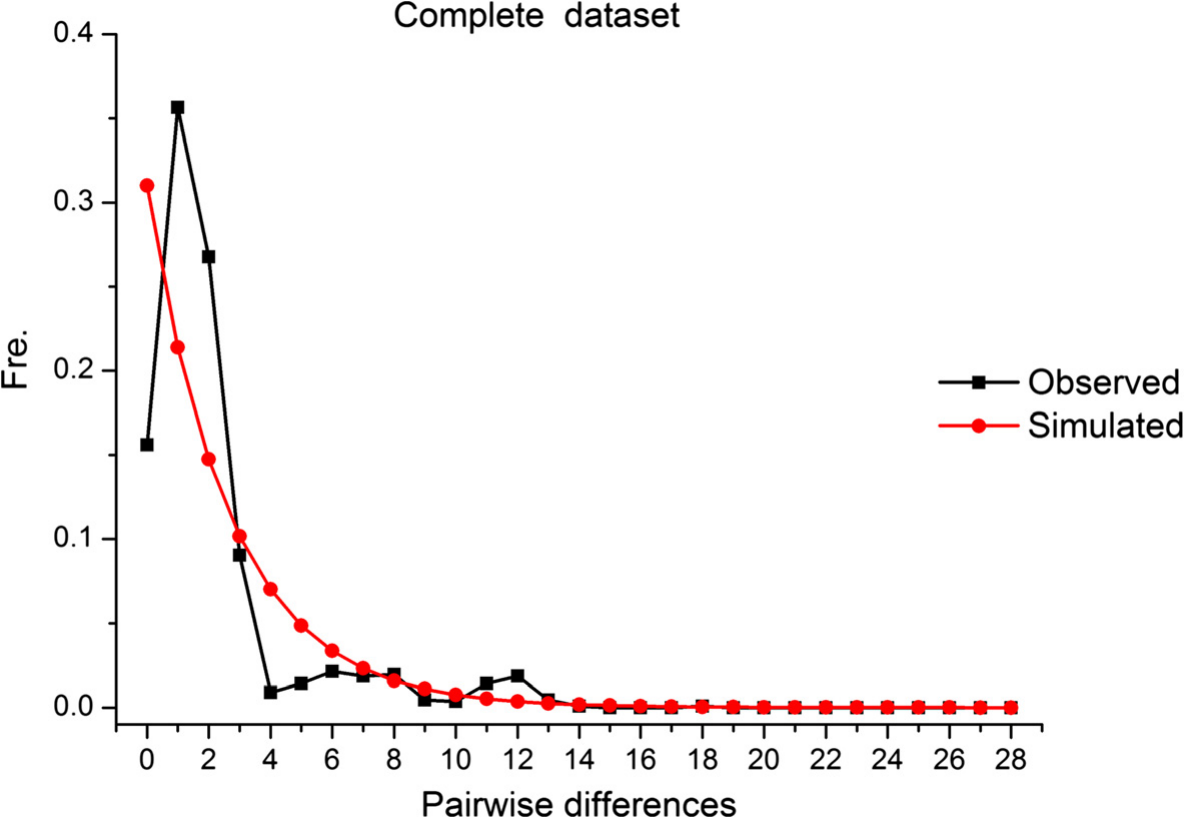
**Mismatch**-**distribution to test the expansion of 48 *B.schroederi *isolates.** The number of nucleotide differences between pairs of sequences is indicated along the x axis, and their frequency along the y axis.

The complete data set of 48 *B*. *schroederi* sequences had a significant, large negative Fu’s *Fs* value (−7.72261, *P* < 0.005). To further explore the demographic history of the *B*. *schroederi* population, the *Fs* statistic values for *Cytb* for the three populations were also estimated. Overall, the *Fs* values were −2.69374 (*P* < 0.05), 1.87372 (*P* > 0.05) and −4.91076 (*P* < 0.005) for the Minshan, Qinling and Qionglai populations, respectively. Mismatch distribution analysis of the complete datasets revealed the presence of a major peak, which suggests that at least one expansion event occurred in the population demographic history of the *B*. *schroederi* population (Figure 4). It is possible that demographic expansions of the parasite occurred after introducing particular individuals into the endemic areas by anthropogenic movements of the giant panda.

## Discussion

In recent years, successive studies employing molecular tools have attempted to understand the genetic diversity, population differentiation, and evolutionary or taxonomic relationships between closely related species. There is no doubt that there has been relatively wide interest in the genetic diversity of parasites. MtDNA markers have higher Fst values than nuclear sequences, and the mtDNA of nematodes evolves more quickly than the mtDNA of other parasites [8,20,21]. Recently, some mt genes (e.g., mt*Cytb*) have been widely applied for analysis of the phylogenetic relationships between nematodes at the subspecies, species, genus and order levels [9,22,23]. Here, the complete *Cytb* gene was sequenced in 48 *B*. *schroederi* isolates sampled from 28 giant pandas inhabiting three different mountain ranges, and the corresponding population genetic diversity of the nematode parasites was subsequently determined.

In general, standard indices of genetic diversity are represented by the number of distinct haplotypes, haplotype diversity (h) and nucleotide diversity (π) [24]. Our results indicated that the three populations of *B*. *schroederi* from the different mountain ranges had a high haplotype diversity and low nucleotide diversity. This phenomenon is frequently observed in a number of other invertebrate animals with large standing population sizes and an extremely high fecundity [25,26], and may reflect the high matrilineal effective population sizes in *B*. *schroederi*, or indicate the occurrence of expansion after a period of low effective population size, as rapid population growth enhances the retention of new mutations.

Fst is appropriate for assessing the levels of differentiation within populations, as genetic drift is assumed to be the major factor that leads to genetic differentiation among closely related populations or over short-term evolution [27]. Our assessment of population genetic structure using the Fst index revealed an absence of significant differentiation among the three *B*. *schroederi* populations (including Qinling mountain), consistent with previous reports based on the ribosome DNA (ITS-1, ITS-2 and 5.8S) [9,28]. Furthermore, the high *Nm* value indicated the occurrence of strong gene flow among the *B*. *schroederi* populations over time. The most likely major contributor to gene flow is the movement of the giant panda. Generally, network approaches are better methods for representing genealogical relationships at a population level than traditional phylogenetic methods, and these approaches take account of several features associated with intraspecific gene evolution, including the persistence of ancestral haplotypes, the existence of multiple descendant haplotypes and often low levels of sequence variation [24]. Based on the network and phylogenetic tree, there was strong support in this study for the existence of a low structure among the *B*. *schroederi* populations from different mountain ranges, in contrast with that reported in giant pandas [10], suggesting absence of significant evolution correlation between *B*. *schroederi* and its host. Additionally, efforts were also made to determine whether a correlation existed between genetic differentiation and geographical distance. The calculated “within population” similarity indices showed that the populations did not follow a pattern of differentiation by distance. Collectively, these results clearly indicate the absence of significant geographical structuring among *B*. *schroederi*. Genetic diversity within populations is affected by their effective population sizes, immigration from other populations and mutation rates, whereas gene flow among populations and the time of divergence of a population from a common ancestral population affect the extent of genetic diversity [29].

In this study, we accounted for differences in sampling effort and the different numbers of individuals sequenced per population. However, it was difficult to obtain sufficient numbers of (sequenced) samples, especially from Qinling Mountains. Although the limit of three populations used for analysis might not be sufficient, strong correlations emerged when the phylogenetic relationship among these haplotypes, even for individual haplotypes (H16) contradicted the general trend (Figure 3). Some *B*. *schroederi* samples from different mountain ranges shared the same haplotypes, indicating that some of the mountain systems have a physical connection, perhaps due to human activity or host movement. Additionally, the haplotype network analysis revealed that the H1 haplotype is the most ancient haplotype (Figure 3), as previously reported ancient haplotypes often have a high frequency and display a trend towards a widespread geographic distribution [30].

Even though the present study was carried out on a small number of populations, genetic diversity was still observable for the parasite. As indicated by the phylogenetic trees (MP and BI) and network, the H8, H14 and H16 haplotypes clearly have a wide distribution, especially the H16 haplotype which could be derivatized by two paths (H1→H5→H16; H1→H14→H16). This phenomenon of genetic diversity in *B*. *schroederi* populations is likely to be influenced by a variety of factors. On the one hand, drainage systems and isolation of the mountain ranges, in particular geographical isolation, have been identified as factors leading to evolutionarily distinct clusters. The Min River valley and the existence of six relatively isolated mountain ranges that giant panda inhabit, three of which were sampled in this study, might result in a distinct population structure among the populations. Second, unlike most free-living organisms, not only the parasites own reproductive and transmission patterns but also the genetics and behavior of the host could influence their genomic variation [31]. Host movement is an important determinant of population genetic structure in parasitic nematodes, and the frequent gene flow and weak population subdivisions will result from mobile vertebrate hosts [32]. The giant panda population has experienced two population expansions, two bottlenecks and two divergences. Evidence indicates that global changes in climate were the primary drivers of population fluctuation for millions of years, and human activities have negatively affected giant pandas for approximately 3,000 years [10]. Previous studies indicated a distinct Qinling Mountain giant panda population [33]. Subsequently, Zhao *et al*. (2012) carried out whole-genome re-sequencing of 34 wild giant pandas to continuously outline the history of the giant panda population, and revealed that the Minshan and Qionglai-Daxiangling-Liangshan populations were also genetically distinct [10]. Prior to the events leading to the divergence of these populations, opportunities for *B*. *schroederi* genetic exchange due to giant panda and, especially, human movements may have independently led to the presence of a large number of haplotypes in the *B*. *schroederi* populations in each area. Indeed, the network map clearly revealed each *B*. *schroederi* population has its own unique haplotypes (Figure 3).

Placing a time scale on molecular data remains a difficult problem, since very little information is available on the *Cytb* gene evolution rate, particularly in parasitic nematodes including ascaridoid species. In the present study, the nematode *H*. *polygyrus* was chosen as a comparison for exploring the evolutionary rate of *Cytb* gene in *B*. *schroederi* due to its known *Cytb* evolution, taxonomic and phylogenetic relationships with *B*. *schroederi*. Based on the molecular evolution rate of *H*. *polygyrus Cytb* gene (3.5-3.7% K_2_P distance per million years) [19], the *B*. *schroederi* divergence time is estimated to be 0.108-0.114 million years (Myr). In addition, it is well-known that sequence diversity can result from an accelerated rate of nucleotide substitution [22]. In our study, the rate of evolution of the *Cytb* gene is approximately 7-fold higher in *B*. *schroederi* than in the giant panda [34]. This result is consistent with phylogenetic studies highlighting the faster molecular evolutionary rate for mtDNA in nematodes, compared with other taxa [34]. A large number of similar studies have analyzed these genes in parasite taxa relative to their hosts [34,35]. In the present study, the 48 complete *Cytb* genes were sequenced and clearly demonstrated the genetic relationships and gene diversity of *B*. *schroederi* populations from different mountain ranges.

## Conclusion

This first investigation of the variability of the complete *Cytb* gene in 48 *B*. *schroederi* isolates sampled from the three main giant panda habitats revealed a lack of population structure (Fst) and a relatively high gene flow for *B*. *schroederi*, and indicates that the low genetic diversity might be a result of either a low DNA mutation rate, frequent movement of the hosts, or a combination of both of these factors. We also presume that *B*. *schroederi* has experienced lineage re-arrangement, as indicated by the high rate of gene flow and lack of a sufficient differentiation period. In addition, expansion phenomena have occurred among the *B*. *schroederi* populations, as most of the genetic variation identified in this study was found within populations, suggesting that a more intensive sampling strategy could possibly uncover the geographic structure of *B*. *schroederi* in more detail.

## Abbreviations

mtCytb: Mitochondrial cytochrome b; ITS1: Internal transcribed spacer region 1; ITS-2: The second internal transcribed spacer; cox1: Cytochrome c oxidase subunit 1; NAD1: NADH dehydrogenase subunit 1; MP: Maximum-parsimony; BI: Bayesian inference; K2P: Kimura 2-parameter; mtDNA: Mitochondrial DNA; Myr: Million years.

## Competing interests

The authors declare that they have no competing interests.

## Authors’ contributions

YGY conceived the concept of the project. ZZH, WCD, GXB, WSX, PXR and YGY collected samples. ZX, XY and SY performed the lab work. ZX and XY carried out the statistical analyses. ZX wrote the initial draft of the manuscript. YGY and XY revised the manuscript. All authors read and approved the final manuscript.

## Supplementary Material

Additional file 1 **Origins and numbers of *B. schroederi *isolates and their hosts.** a, GenBank accession numbers of mitochondrial *Cytb* sequences of these *B*. *schroederi* samples. Click here for file

Additional file 2 **Polymorphic sites in complete mt*Cytb *gene.** The nucleotides present at each variable site among the 48 *B*. *schroederi* isolates. Click here for file
